# Burden of hospitalizations and outpatient visits associated with moderate and severe acute graft-versus-host disease in Finland and Sweden: a real-world data study

**DOI:** 10.1007/s00520-022-06915-9

**Published:** 2022-03-02

**Authors:** Lorenzo Sabatelli, Mikko Keränen, Elisabet Viayna, Montserrat Roset, Nuria Lara, Daniel Thunström, Minja Pfeiffer, Malin Nicklasson, Maija Itälä-Remes

**Affiliations:** 1 Incyte Biosciences International Sàrl, Rue Docteur-Yersin 10, 1110 Morges, Switzerland; 2 Helsinki University Hospital, Helsinki, Finland; 3 IQVIA Real World Solutions, Barcelona, Spain; 4 Department of Hematology and Coagulation, Sahlgrenska Academy, University of Gothenburg, Gothenburg, Sweden; 5 Turku University Hospital, Turku, Finland

**Keywords:** Acute graft-versus-host disease, Outpatient, Hospitalization, Real-world data, Severity grades, MAGIC, Glucksberg

## Abstract

**Purpose:**

The aim of this study was to describe patient characteristics and quantify hospital stays and outpatient visits (H&OV) following diagnosis with moderate-to-severe acute graft-versus-host disease (aGVHD) in Finland and Sweden.

**Methods:**

A retrospective chart audit collected data from patient medical records of 3 specialized centers performing allogeneic hematopoietic stem cell transplantation (HSCT; Finland, *n* = 2; Sweden, *n* = 1). Eligible patients received allogeneic HSCT (January 1, 2016–June 30, 2017) from any donor source, were diagnosed with grade II–IV aGVHD (MAGIC or modified Glucksberg criteria) at any time from transplantation to 12 months before data collection, and were ≥ 18 years old at diagnosis. Criteria for comparing patients graded with modified Glucksberg and MAGIC severity scales were defined.

**Results:**

Fifty-five patients (Finland, *n* = 45; Sweden, *n* = 10) were included. Myeloablative conditioning was the most common conditioning regimen (81.8%); immunosuppression regimens were based on combinations of methotrexate (96.4%), in vivo T-cell depletion (80.0%), cyclosporine (63.6%), mycophenolate (40.0%), and tacrolimus (34.5%). Sixteen patients (29.1%) developed grade III/IV aGVHD; skin was the most common organ involved (80.0%). Most patients required ≥ 1 hospital stay (89.1%; median of 2 hospitalizations per patient); 7 patients (14.3%) required admission to an intensive care unit. Median hospitalization duration from HSCT to discharge was 26 days. Most patients also required outpatient or emergency department visits (90.9%). Subgroup analyses showed longer hospital stays for patients receiving multiple treatment lines; no clear differences in H&OV were observed between prophylactic regimens.

**Conclusion:**

Based on this retrospective study, moderate-to-severe aGVHD is associated with considerable healthcare resource utilization in Finland and Sweden, particularly in patients who received multiple lines of therapy.

**Supplementary Information:**

The online version contains supplementary material available at 10.1007/s00520-022-06915-9.

## Introduction

Graft-versus-host disease (GVHD), a serious complication of allogeneic hematopoietic stem cell transplantation (HSCT), is a clinical syndrome caused by the response of alloreactive donor T cells to histocompatibility antigens expressed on tissues of the transplant recipient [1, 2]. The European Society for Blood and Marrow Transplantation and National Institutes of Health Center for International Blood and Marrow Transplant Research (EBMT–NIH-CIBMTR) joint classification for acute GVHD (aGVHD) includes classic aGVHD, defined by the occurrence of aGVHD manifestations within 100 days after transplantation or donor lymphocyte infusion (DLI), as well as persistent, recurrent, or late-onset forms of aGVHD, which occur beyond 100 days posttransplantation or after DLI [3, 4]. Clinical manifestations of aGVHD typically develop in the skin, gastrointestinal (GI) tract, or liver, leading to erythema, maculopapular rash, nausea, vomiting, anorexia, diarrhea, ileus, increase of liver transaminases, or cholestatic hyperbilirubinemia; severity of aGVHD is determined by the extent of involvement of these principal target organs [2, 4, 5].

Despite routine use of prophylactic regimens, aGVHD occurs in 30 to 60% of patients undergoing allogeneic HSCT [6, 7]. Corticosteroids are currently a standard of care for first-line therapy for aGVHD. However, up to 60% of patients do not respond adequately to steroids [8–11]. For these patients, a choice of second-line therapy remains controversial.

Acute GVHD is a leading cause of post-HSCT nonrelapse mortality and has been previously associated with increased hospital stays and outpatient visits (H&OV). However, real-world data (RWD) for aGVHD-related outcomes of transplanted patients and the associated H&OV are scarce and partly outdated. A retrospective analysis of patients undergoing allogeneic HSCT between 2006 and 2009 in the UK showed significantly higher rates of hospital readmission leading to higher costs for patients with GVHD compared to those without [12]. Furthermore, retrospective analyses from large US hospitals have shown that patients who developed aGVHD, especially in the subgroups of steroid-refractory or high-risk disease, had significantly longer hospital stays, higher rates of hospital readmissions, higher intensive care unit (ICU) admission rates, greater costs, and increased risk of mortality compared with those who did not develop GVHD [13–15].

Currently, there are only a few studies reporting aGVHD-related morbidity and mortality or H&OV-related healthcare resource utilization in contemporary European transplantation centers [16, 17]; thus, RWD analyses from additional sample populations in European countries are needed to more precisely determine GVHD-related burden. The aim of this study was to describe the clinical presentation, prophylactic treatments, hospitalizations, and outpatient visits among patients who developed moderate or severe aGVHD.

## Methods

### Study design and patients

This was a noninterventional, retrospective chart review study that originally planned to enroll patients in 4 European countries (Germany, Italy, Sweden, and Finland); however, due to the sponsor’s decision to reduce the scope of the study for resource considerations, study data were ultimately retrieved from 2 sites in Finland (Turku and Helsinki) and one site in Sweden (Gothenburg). The sites were specialized centers belonging to the EBMT that routinely perform allogeneic HSCT. Patients were included retrospectively and consecutively, starting with those who received HSCT on June 30, 2017, and subsequently developed aGVHD, and working backward recruiting those who had received HSCT until January 1, 2016, or until the target sample size of approximately 4 to 25 patients per center had been reached, whichever occurred first (Online Resource 1). Patient charts were reviewed from the index date (date of allogeneic HSCT) until the day of data collection, death, or loss to follow-up, whichever occurred first.

Study eligibility criteria included receipt of a first allogeneic HSCT between January 1, 2016, and June 30, 2017, from any donor source using bone marrow, peripheral blood stem cells (PBSCs), or umbilical cord blood; diagnosis of grade II–IV aGVHD based on Mount Sinai Acute GVHD International Consortium (MAGIC) criteria [18] (or alternatively, a II–IV severity grade per the Glucksberg Severity Index or the Keystone Criteria [19], or grade B–D according to International Blood and Marrow Transplant Research (IBMTR) criteria [20]) any time from transplantation to 12 months before data collection; and age ≥ 18 years at the time of aGVHD diagnosis. Only patients with complete clinical records containing the main clinical characteristics related to the original disease requiring allogeneic HSCT and clinical information on aGVHD presentation and treatment were included. Exclusion criteria included receipt of > 1 HSCT; participation in a GVHD prophylaxis trial with a primary completion date later than December 31, 2018 (to ensure that trial results would be available by the time of patient enrollment), or in any GVHD treatment trial at any point during the data collection period (i.e., January 2016 until the time of data collection, death, or loss to follow-up); disease progression before the first aGVHD episode; or aGVHD following DLI.

### Data collection

Patient data were collected from patient medical records and entered into electronic case report forms (eCRFs). Data from the eCRFs corresponding to eligibility criteria were regularly reviewed to ensure inclusion of eligible patients only and for consistency. Data on patient demographics, transplant characteristics, disease risk index (DRI; an index for stratification of patients undergoing HSCT by disease risk) [21, 22], aGVHD clinical characteristics, treatments, outcomes, and H&OV (hospitalizations/inpatient admissions and outpatient and emergency department visits) were collected. For each patient, length of hospitalizations and ICU stays were calculated based on date of admission and discharge; only hospitalizations and ICU stays that took place during or after aGVHD diagnosis were considered. If a patient was in the hospital at the time of aGVHD diagnosis, the ongoing hospitalization episode was included in the analysis, but any days spent in the hospital before the diagnosis were not considered.

Transplant conditioning regimens were recorded and classified as myeloablative conditioning (MAC), reduced-intensity conditioning (RIC), and sequential conditioning. Prophylactic regimen categories included ex vivo T-cell depletion, in vivo T-cell depletion (antithymocyte globulin [ATG], alemtuzumab, other), cyclosporine, steroid, tacrolimus, posttransplant cyclophosphamide, methotrexate, mycophenolate, sirolimus, and other. Data were collected on the number of treatment lines initiated.

### Statistical analyses

All disease diagnoses (e.g., comorbidities) and medical procedure terms were recorded and coded using the Medical Dictionary for Regulatory Activities. All computations were performed using SAS® version 9.2 or higher (SAS Institute, Cary, NC, USA). To ensure comparability across different grading systems, aGVHD severity was compared across scales and based on extent of skin, liver, and GI involvement, with grades II–IV (MAGIC and modified Glucksberg/Keystone criteria) defined as skin stage ≥ 3 and/or liver ≥ 1 and/or GI ≥ 1, and grades B–D (IBMTR criteria) defined as skin stage ≥ 2 and/or liver ≥ 1 and/or GI ≥ 1 (Online Resources 2A and B) [4]. Mapping rules to compare patients graded per MAGIC criteria with those graded using a different scale were derived from grade and organ score definitions specific to each scale (Online Resources 3 and 4).

Data were summarized using descriptive statistics. Continuous variables were reported using mean, median, standard deviation, interquartile range, minimum, and maximum. Categorical values were summarized as number and proportion of the total study population and by subgroups, where appropriate. Missing values were reported for categorical and continuous values but were excluded to calculate percentages of patients. Although the study has a descriptive design, the probability that subgroup differences in outcomes (large or larger than observed) could have occurred under the null hypothesis of no difference was calculated using *P*-values, if ≥ 10 patients per category were reached. No *P*-value threshold was prespecified or used to draw conclusions. The chi-squared test was used to compare categorical variables, and the nonparametric Kruskal–Wallis test was used to compare continuous variables.

Subgroup analyses were conducted to evaluate differences in H&OV (e.g., number and length of hospitalizations, reasons for hospitalizations, number and length of ICU admissions, number of and reasons for emergency department and outpatient visits) based on number of treatment lines (1 vs ≥ 2 lines of treatment) and type of prophylactic regimens received (1 tacrolimus plus mycophenolate, in vivo T-cell depletion and methotrexate; 2 in vivo T-cell depletion plus methotrexate and cyclosporine; 3 methotrexate plus cyclosporine; or 4 other). A change in treatment lines was defined as replacement of an anti-aGVHD drug with another anti-aGVHD drug and/or addition of an anti-aGVHD drug to the previous regimen.

## Results

### Patient characteristics

Overall, there were approximately 50 patients in Finland and 80 patients in Sweden who developed grade II to IV aGVHD after HSCT from January 1, 2016, to June 30, 2017 (calculation based on EBMT data [23]). A total of 55 patients (Finland, *n* = 45; Sweden, *n* = 10) were treated in participating centers, met inclusion criteria, and were therefore included in this study (Table 1). Acute myeloid leukemia was the most common indication for HSCT (*n* = 19 [34.5%]), followed by multiple myeloma (*n* = 8 [14.5%]). At the time of HSCT, most patients were in complete (*n* = 34 [61.8%]) or partial (*n* = 12 [21.8%]) remission, and the most common DRI was intermediate (*n* = 27 [49.1%]). Most donors were unrelated (*n* = 42 [76.4%]), and of these, only 2 patients (4.8%) received a human leukocyte antigen-mismatched graft. Most patients received a PBSC graft (*n* = 53 [96.4%]). All patients for whom chimerism was determined (*n* = 36) reached full donor chimerism as their maximum level of chimerism.

**Table 1:** Patient demographics and clinical characteristics at transplant

Characteristic	Finland (*n* = 45)	Sweden (*n* = 10)	Total (*N* = 55)
Age at HSCT, *y*			
Median (range)	54.0 (21.0–66.0)	44.5 (20.0–71.0)	51.0 (20.0–71.0)
Age at aGVHD diagnosis, *y*			
Median (range)	54.0 (21.0–66.0)	44.5 (20.0–71.0)	51.0 (20.0–71.0)
Male, *n* (%)	25 (55.6)	5 (50.0)	30 (54.5)
Primary disease diagnosis, *n* (%)			
AML	16 (35.6)	3 (30.0)	19 (34.5)
Multiple myeloma	8 (17.8)	0	8 (14.5)
B-cell lymphoma (NHL)	3 (6.7)	2 (20.0)	5 (9.1)
MDS	4 (8.9)	0	4 (7.3)
MPN	4 (8.9)	1 (10.0)	5 (9.1)
ALL	3 (6.7)	1 (10.0)	4 (7.3)
Hodgkin lymphoma	3 (6.7)	1 (10.0)	4 (7.3)
Other^a^	3 (6.7)	2 (20.0)	5 (9.1)
Stage at transplant, *n* (%)			
Complete remission	29 (64.4)	5 (50.0)	34 (61.8)
Partial remission	9 (20.0)	3 (30.0)	12 (21.8)
Active relapse or PD	5 (11.1)	1 (10.0)	6 (10.9)
Untreated	2 (4.4)	1 (10.0)	3 (5.5)
DRI,^b^ *n* (%)			
Low	8 (17.8)	2 (20.0)	10 (18.2)
Intermediate	21 (46.7)	6 (60.0)	27 (49.1)
High	9 (20.0)	2 (20.0)	11 (20.0)
Very high	1 (2.2)	0	1 (1.8)
Unknown	6 (13.3)	0	6 (10.9)
Stem cell source, *n* (%)			
PBSC	45 (100.0)	8 (80.0)	53 (96.4)
Bone marrow	0	2 (20.0)	2 (3.6)
Related donor, *n* (%)	8 (17.8)	5 (50.0)	13 (23.6)
Fully HLA-matched twin	5 (62.5)	0	5 (38.5)
HLA-mismatched related donor	0	1 (20.0)	1 (7.7)
HLA-matched related donor	3 (37.5)	4 (80.0)	7 (53.8)
Unrelated donor, *n* (%)	37 (82.2)	5 (50.0)	42 (76.4)
HLA matched	35 (94.6)	5 (100.0)	40 (95.2)
HLA mismatched	2 (5.4)	0	2 (4.8)
Recipient serologic CMV-positive status, *n* (%)	29 (64.4)	8 (80.0)	37 (67.3)
Maximum level of chimerism, *n* (%)			
Full donor chimerism	26 (57.8)	10 (100.0)	36 (65.5)
Mixed or partial chimerism after reaching full chimerism^c^	5 (19.2)	7 (70.0)	12 (33.3)
Unknown	19 (42.2)	0	19 (34.5)
aGVHD organ symptom involvement, *n* (%)			
Skin	37 (82.2)	7 (70.0)	44 (80.0)
Lower GI tract	23 (51.1)	4 (40.0)	27 (49.1)
Liver	11 (24.4)	1 (10.0)	12 (21.8)
Upper GI tract	10 (22.2)	0	10 (18.2)

*aGVHD*, acute graft-versus-host disease; *ALL*, acute lymphocytic leukemia; *AML*, acute myeloid leukemia; *CMV*, cytomegalovirus; *DRI*, disease risk index; *GI*, gastrointestinal; *HSCT*, hematopoietic stem cell transplantation; *HLA*, human leukocyte antigen; *MDS*, myelodysplastic syndrome; *MPN*, myeloproliferative neoplasm; *NHL*, non-Hodgkin lymphoma; *PBSC*, peripheral blood stem cell; *PD*, progressive disease. ^a^ “Other” includes chronic myeloid leukemia (*n* = 2), blastic plasmacytoid dendritic cell neoplasia, chronic myelomonocytic leukemia, and T-cell lymphoma (*n* = 1 each). ^b^DRI determined as described in Armand P, et al. *Blood*. 2014;123(23):3664–3671. ^c^Among patients who reached full chimerism (Finland, *n* = 26; Sweden, *n* = 10; total, *n* = 36)

### Transplant conditioning regimen and aGVHD prophylaxis

**Fig. 1:**
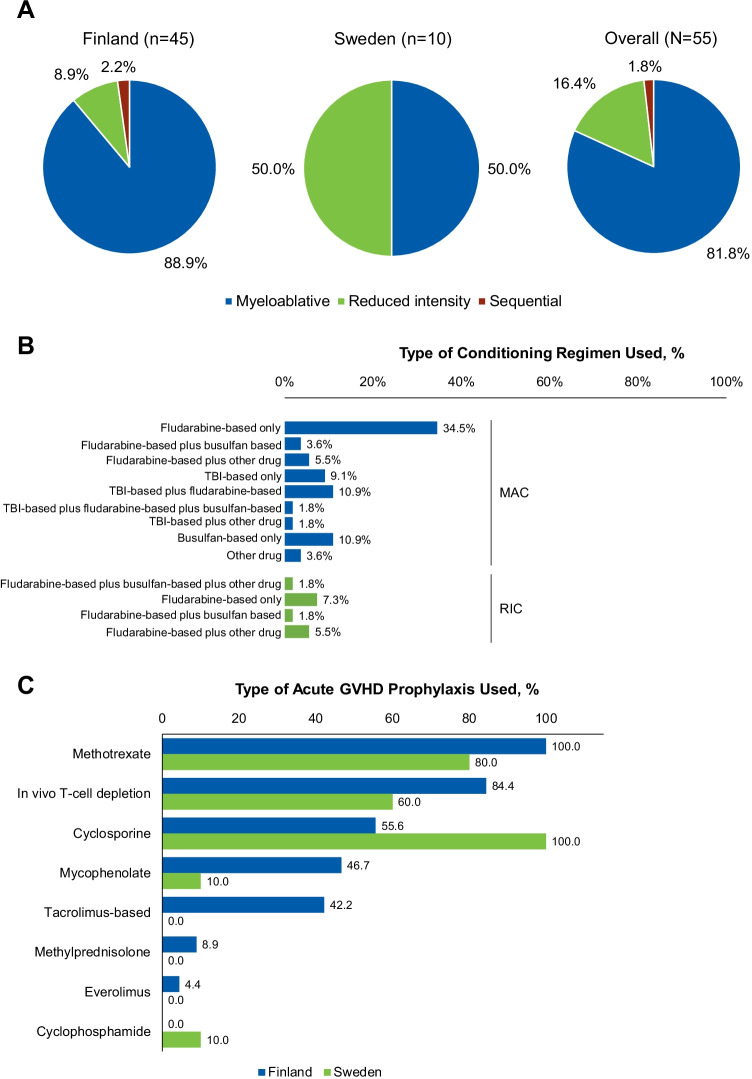
Transplant conditioning regimen and aGVHD prophylaxis. **A** Type of conditioning regimen used, by country and overall. **B** Specific conditioning regimens by category (MAC or RIC). **C** aGVHD prophylaxis used by country. aGVHD, acute graft-versus-host disease; MAC, myeloablative conditioning; RIC, reduced-intensity conditioning; TBI, total body irradiation

Myeloablative conditioning was the most common transplant conditioning regimen and was used in 45 transplants (81.8%), followed by RIC in 9 transplants (16.4%; Fig. 1A). Most patients received fludarabine-based conditioning (*n* = 41/55 [74.5%]), and 15 (27.3%) patients received total body irradiation (TBI)-based conditioning (Fig. 1B). Fludarabine was most frequently administered alone (*n* = 26/41; 63.4%), followed by combinations with TBI (*n* = 8/41 [19.5%]), thiotepa (*n* = 4/41 [thiotepa alone, *n* = 1; thiotepa plus cyclophosphamide, *n* = 3]; 9.8%), treosulfan (*n* = 2/41 [4.9%]), or melphalan (*n* = 1/41 [2.4%]). Busulfan administered over 3 or 4 days was defined as MAC, and over 2 days as RIC (the total of daily doses was the same). Treosulfan was administered over 3 days; a daily dose of 14 g/m^2^ was classified as MAC, and a daily dose of 10 g/m^2^ as RIC.

Immunosuppression was mainly based on the calcineurin inhibitors cyclosporine (*n* = 35/55 [63.6%]) and tacrolimus (*n* = 19/55 [34.5%]). Short-course methotrexate was used in almost all transplants (*n* = 53/55 [96.4%]), and a majority of patients also received ATG as in vivo T-cell depletion (*n* = 44/55 [80.0%]). Mycophenolate mofetil was frequently added to the combination (*n* = 22/55 [40.0%]; Fig. 1C).

The most frequent prophylaxis combination was cyclosporine, methotrexate, and ATG (*n* = 20/55 [36.4%]), followed by tacrolimus, methotrexate, ATG, and mycophenolate (*n* = 14/55 [25.5%]) and cyclosporine with methotrexate (*n* = 7/55 [12.7%]).

### Acute GVHD severity and organ symptom involvement

The scales used for grading aGVHD severity across the 3 participating centers were MAGIC (*n* = 29/55 [52.7%]) or modified Glucksberg (*n* = 26/55 [47.3%]; Online Resource 5). Thirty-nine patients (70.9%) and 16 patients (29.1%) developed grade II and grades III/IV aGVHD, respectively. Skin was the most common organ involved (*n* = 44/55 [80.0%]), followed by the lower GI tract (*n* = 27/55 [49.1%]), liver (*n* = 12/55 [21.8%]), and upper GI tract (*n* = 10/55 [18.2%]; Table 1).

### Nonpharmacologic H&OV since aGVHD diagnosis

**Fig. 2:**
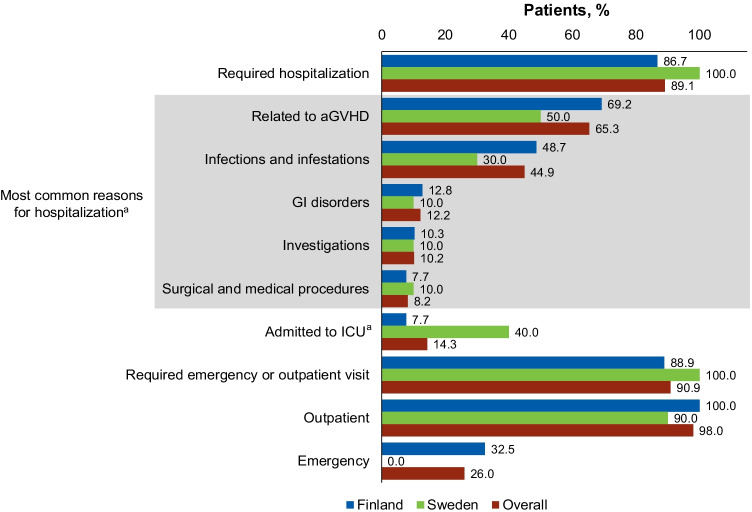
H&OV: hospitalizations and outpatient and emergency department visits. ^a^Percentages were calculated over the number of hospitalized patients and not over the whole study sample. aGVHD, acute graft-versus-host disease; GI, gastrointestinal; H&OV, hospital stays and outpatient visits; ICU, intensive care unit

Most patients with aGVHD (*n* = 49/55 [89.1%]) required at least one hospitalization period, primarily due to aGVHD (*n* = 32/49 [65.3%]) or infections/infestations (*n* = 22/49 [44.9%]; Fig. 2). Median (range) number of hospitalization periods per patient was 2.0 (0.0–10.0). Seven patients (*n* = 7/49 [14.3%]) required admission to an ICU (Fig. 2). From the date of HSCT to discharge during the initial transplant period, the median duration of hospitalization was 26.0 days (Table 2), and nearly half of all hospitalizations lasted > 7 days (*n* = 72/158 [45.6%]; Online Resource 6). Mean (SD) days spent in the hospital and ICU following aGVHD grade II–IV diagnosis per 100 days of observation were 17.9 (31.4) and 0.7 (2.7), respectively (Table 2). In addition, most patients required an outpatient or emergency department visit following aGVHD grade II–IV diagnosis (*n* = 50/55 [90.9%]; Fig. 2). On average, patients required a mean (SD) 11.7 (11.1) outpatient and 0.3 (0.6) emergency visits per year (Table 2).

**Table 2:** Hospitalizations

	**Finland (*n* = 45)**	**Sweden (*n* = 10)**	**Total (*N* = 55)**
Number of hospitalizations per patient			
Mean (SD)	2.9 (2.8)	3.0 (1.7)	2.9 (2.7)
Median (range)	2.0 (0.0–10.0)	2.5 (1.0–6.0)	2.0 (0.0–10.0)
Total days of hospitalization per patient^a^			
Mean (SD)	31.8 (30.8)	83.0 (79.8)	41.3 (47.5)
Median (range)	25.5 (0.0–137.0)	47.0 (6.0–214.0)	26.0 (0.0–214.0)
Days of hospitalization/100 patient-days^a^			
Mean (SD)	15.4 (30.1)	29.2 (36.2)	17.9 (31.4)
Median (range)	3.6 (0.0–100.0)	12.9 (0.5–86.7)	3.9 (0.0–100.0)
Days of ICU stay			
Mean (SD)	0.5 (2.2)	4.1 (8.8)	1.1 (4.3)
Median (range)	0.0 (0.0–14.0)	0.0 (0.0–28.0)	0.0 (0.0–28.0)
Days of ICU stay/100 patient-days			
Mean (SD)	0.5 (2.3)	1.8 (3.8)	0.7 (2.7)
Median (range)	0.0 (0.0–13.5)	0.0 (0.0–12.3)	0.0 (0.0–13.5)
Number of outpatient visits per patient-year, mean (SD)	9.3 (6.4)	23.7 (19.8)	11.7 (11.1)
Number of emergency visits per patient-year, mean (SD)	0.3 (0.6)	0	0.3 (0.6)

*ICU*, intensive care unit. ^a^Calculated based on number of patients requiring hospitalization (Finland, *n* = 44; Sweden, *n* = 10; total, *n* = 54)

### Subgroup analyses

A subgroup analysis evaluating H&OV by number of treatment lines for aGVHD demonstrated similar rates of hospitalization between patients who received one treatment line (*n* = 22/24 [91.7%]) and those who needed at least 2 treatment lines (*n* = 27/31 [87.1%]). Patients who received at least 2 lines of therapy had a median hospitalization duration of 6.1 days per 100 patient-days, and those who received only one treatment line had 1.9 days (≥ 2 lines vs 1 line, *P* = 0.03; Table 3). Median number of hospitalization periods per patient and median number of days at ICU per 100 patient-days were the same between patients who received ≥ 2 lines or 1 line of treatment. The proportion of the study population requiring outpatient or emergency department visits was 87.1% among patients with ≥ 2 lines of prior treatment and 95.8% among those receiving only one prior line of treatment. The mean number of outpatient visits per person per year was similar for the 2 subgroups (≥ 2 lines, 11.4; 1 line, 12.0; *P* = 0.16). Mean number of emergency department visits per patient-year was two-fold higher for patients who received only 1 line of treatment compared with those receiving 2 or more lines (≥ 2 lines, 0.2 visits/patient-year; 1 line, 0.4 visits/patient-year; *P* = 0.16); however, it cannot be excluded that this may be due only to chance.

**Table 3:** Subgroup analysis of H&OV by prior lines of treatment

	**1 line (*n* = 24)**	** ≥ 2 lines (*n* = 31)**	***P*-value**	**Total (*N* = 55)**
Required hospitalization, n (%)	22 (91.7)	27 (87.1)		49 (89.1)
Total number of hospitalizations	55	104		159
Number of hospitalizations per patient				
Mean (SD)	2.3 (2.1)	3.4 (3.0)	0.55	2.9 (2.7)
Median (range)	2.0 (0.0–10.0)	2.0 (0.0–10.0)		2.0 (0.0–10.0)
Duration of hospitalization per patient, days				
Mean (SD)	22.2 (21.0)	56.5 (56.8)		41.3 (47.5)
Median (range)	14.5 (0.0–81.0)	35.0 (0.0–214.0)		26.0 (0.0–214.0)
Days of hospitalization/100 patient-days				
Mean (SD)	7.4 (19.8)	26.4 (36.4)	0.03	17.9 (31.4)
Median (range)	1.9 (0.0–98.4)	6.1 (0.0–100.0)		3.9 (0.0–100.0)
Required admission to ICU, *n* (%)^a^	1 (4.5)	6 (22.2)		7 (14.3)
Duration of ICU stay per patient, days				
Mean (SD)	0.6 (2.9)	1.6 (5.2)		1.1 (4.3)
Median (range)	0.0 (0.0–14.0)	0.0 (0.0–28.0)		0.0 (0.0–28.0)
Days of ICU stay/100 patient-days				
Mean (SD)	0.3 (1.7)	1.0 (3.2)	0.50	0.7 (2.7)
Median (range)	0.0 (0.0–8.2)	0.0 (0.0–13.5)		0.0 (0.0–13.5)
Required outpatient or emergency department visit, *n* (%)	23 (95.8)	27 (87.1)		50 (90.9)
Type of visit, *n* (%)				
Outpatient	23 (100.0)	26 (96.3)		49 (98.0)
Emergency	8 (34.8)	5 (18.5)		13 (26.0)
Missing	0	1 (3.7)		1 (2.0)
Number of outpatient visits per patient-year, mean (SD)^b^	12.0 (7.9)	11.4 (13.2)	0.16	11.7 (11.1)
Number of emergency department visits per patient-year, mean (SD)^b^	0.4 (0.7)	0.2 (0.4)	0.16	0.3 (0.6)

*H&OV*, hospital stays and outpatient visits; *ICU*, intensive care unit. ^a^Percentages calculated based on number of patients requiring hospitalization (1 line, *n* = 22; ≥ 2 lines, *n* = 27; total, *n* = 49). ^b^Includes patients with nonmissing visit type information (1 line, *n* = 24; ≥ 2 lines, *n* = 30; total, *n* = 54)

When H&OV was assessed by prophylactic regimen categories, the lowest rate of hospitalization was observed among patients who received tacrolimus plus mycophenolate, in vivo T-cell depletion, and methotrexate (71.4% vs 100.0% for in vivo T-cell depletion plus methotrexate and cyclosporine, 100.0% for methotrexate plus cyclosporine, and 85.7% for other regimens). Patients who received tacrolimus plus mycophenolate, in vivo T-cell depletion, and methotrexate also spent considerably less (about half as many) days in the hospital on average (9.8 per 100 patient-days) compared with other treatment regimens. However, it cannot be ruled out that the observed differences may be due only to chance (Table 4).

**Table 4:** Subgroup analysis of H&OV by prophylactic regimen

	**Tacrolimus, mycophenolate, in vivo T-cell depletion, methotrexate (*n* = 14)**	**In vivo T-cell depletion, methotrexate, cyclosporine (*n* = 20)**	**Methotrexate, cyclosporine (*n* = 7)**	**Other (*n* = 14)**	***P*-value**
Required hospitalization, *n* (%)	10 (71.4)	20 (100.0)	7 (100.0)	12 (85.7)	
Total number of hospitalizations	46	64	15	34	
Number of hospitalizations per patient					
Mean (SD)	3.3 (3.4)	3.2 (2.6)	2.1 (1.9)	2.4 (2.3)	0.77
Median (range)	3.0 (0.0–10.0)	2.0 (1.0–10.0)	1.0 (1.0–6.0)	2.0 (0.0–9.0)	
Duration of hospitalization per patient, days					
Mean (SD)	33.1 (34.9)	51.9 (52.1)	24.3 (21.3)	43.1 (58.3)	
Median (range)	28.0 (0.0–104.0)	28.0 (8.0–179.0)	17.0 (6.0–69.0)	25.5 (0.0–214.0)	
Days of hospitalization/100 patient-days					
Mean (SD)	9.8 (23.9)	19.6 (29.8)	18.3 (36.6)	22.8 (37.9)	0.34
Median (range)	3.8 (0.0–91.9)	6.4 (1.2–97.9)	1.7 (0.5–100.0)	3.1 (0.0–98.4)	
Required admission to ICU, *n* (%)^a^	1 (10.0)	4 (20.0)	1 (14.3)	1 (8.3)	
Duration of ICU stay per patient, days					
Mean (SD)	0.4 (1.3)	2.7 (6.9)	0.4 (1.1)	0.1 (0.5)	
Median (range)	0.0 (0.0–5.0)	0.0 (0.0–28.0)	0.0 (0.0–3.0)	0.0 (0.0–2.0)	
Days of ICU stay/100 patient-days)					
Mean (SD)	1.0 (3.6)	1.2 (3.3)	0.1 (0.3)	0.1 (0.4)	0.65
Median (range)	0.0 (0.0–13.5)	0.0 (0.0–12.3)	0.0 (0.0–0.8)	0.0 (0.0–1.6)	

*H&OV*, hospital stays and outpatient visits; *ICU*, intensive care unit. ^a^Percentages calculated based on number of patients requiring hospitalization (tacrolimus, mycophenolate, in vivo T-cell depletion, methotrexate, *n* = 10; in vivo T-cell depletion, methotrexate, cyclosporine, *n* = 20; methotrexate, cyclosporine, *n* = 7; other, *n* = 12)

### Mortality rates since aGVHD development

At 6 months after aGVHD diagnosis, mortality rates in Finland and Sweden were 17.8% (*n* = 8) and 10.0% (*n* = 1), respectively. At 12 months, the mortality rates were 17.8% (*n* = 8) and 40.0% (*n* = 4).

## Discussion

The present study provides a detailed description of the clinical characteristics and aGVHD-related H&OV of patients who developed aGVHD after allogeneic HSCT. These RWD were collected from 2 reference transplantation centers in Finland and one in Sweden. Owing to data availability, precise coverage of the Swedish population was unknown because exact estimates were not available; however, the study had a high coverage in the Finnish population, with 80 to 90% of nationwide eligible patients being included in this chart review study [23].

Although fewer than 30% of patients in the study presented with grade III or IV aGVHD, nearly 90% of patients required hospitalization. Overall, patients were hospitalized for a median of 26 days after a diagnosis of aGVHD and required more than 10 outpatient or emergency department visits per year. It should be noted that the hospitalization numbers in Gothenburg (Sweden) were higher than those reported in the 2 Finnish sites; one possible reason for this discrepancy is that it is standard at Gothenburg to call for multiple follow-up visits during the first few months after HSCT, with additional visits required following aGVHD diagnosis, which may result in hospital admissions. When stratified by treatment lines, patients who received at least 2 treatment lines had a longer hospital stay compared with those who received only one treatment line, although the rate of ICU admissions or length of treatment at ICU did not differ between these subgroups. With the exception of patients who received tacrolimus plus mycophenolate, in vivo T-cell depletion, and methotrexate, most prophylaxis regimens resulted in similar rates and duration of hospitalization or ICU admissions. These findings are consistent with those of the few other available studies conducted in the USA. An analysis of a large data set of hospital discharges showed that patients diagnosed with aGVHD had a significantly longer length of stay during initial hospitalization for HSCT versus those without aGVHD (31 vs 24 days, respectively) and were more likely to require ICU admission (40.6% vs 25.4%) [13]. Another national analysis of inpatient discharge records similarly showed an increased length of hospital stay among patients who developed aGVHD after HSCT versus those who did not (42.0 vs 26.0 days, respectively), as well as increased in-hospital mortality rates (16.2% vs 5.3%) [14].

These study’s findings show that, besides requiring considerable medical attention and arguably competing with other health conditions for limited healthcare resources, aGVHD is associated with a substantial financial burden for healthcare payers. In fact, based on the average cost of a day of hospitalization in Finland and Sweden, and on this study’s results, the average cost per patient for aGVHD-associated hospitalizations would amount to ~ $25,000–$40,000 USD and ~ $90,000 USD, respectively; similarly, based on the average cost of an outpatient visit in Finland and Sweden, the cost per patient for aGVHD-related outpatient visits per year amounts to ~ $600–$1700 USD and ~ $4700 USD, respectively (Online Resource 8). Although referring to a very different healthcare environment, these figures are overall comparable to those provided by a large US healthcare claims database study, which showed that the total healthcare costs incurred during a 1-year period following allogeneic HSCT were $100,000 USD higher for patients who developed aGVHD; additionally, hospital length of stay was nearly 3 weeks longer than for those without aGVHD [15]. Therefore, the present study supports and reinforces the findings of the few recent (US-based) studies, providing novel figures specific to the burden of H&OV in Finland and Sweden.

Limitations of this study include those typical of a retrospective chart review, such as the potential for medical charts being incomplete or inaccurate. These potential issues may have affected the calculations of H&OV and hospitalization durations, as well as information on treatment and prophylaxis. Nonetheless, the chart abstraction was conducted by qualified investigators from the enrolled centers familiar with the local ways of recording medical information. Variables collected were standard and clinically meaningful within acute GVHD populations, and specific information on variable definition was collected when needed. The use of an electronic case report made possible the implementation of an algorithm that performed consistency checks across the data filled in for each patient, reducing the risk of reporting errors. Finally, additional checks and triangulation of the clinical information were performed during the analyses.

The differences in aGVHD organ staging and grading systems (i.e., MAGIC vs modified Glucksberg), which could have an impact on the interpretation of results, were addressed by grouping and restaging modified Glucksberg cases using MAGIC criteria, instead of doing the opposite mapping (which would be affected by considerably higher uncertainty due to stage and grade definitions in the 2 scales) or naively comparing patients graded in MAGIC with patients graded in Glucksberg, as explained in Online Resource 7. In addition, the study was also limited by the small sample size, limiting statistical comparisons, especially at the subgroup level and particularly for Sweden, where information was only available from one hospital. Finally, variability existed between the Finland and Sweden data sets in terms of patient selection, which may have also affected the calculations and findings of the study.

In conclusion, findings from this study show that moderate to severe aGVHD is associated with considerable H&OV in Finland and Sweden, particularly in patients who received multiple lines of therapy. Larger follow-up studies across multiple regions, including prospective analyses, should be conducted to assess the generalizability of these findings to the aGVHD patient population as a whole.

## Supplementary Information

Below is the link to the electronic supplementary material.

Supplementary file1 (DOCX 919 KB)

## Data Availability

Access to individual patient-level data is not available for this study. Information on Incyte’s clinical trial data sharing policy and instructions for submitting clinical trial data requests are available at: https://www.incyte.com/Portals/0/Assets/Compliance%20and%20Transparency/clinical-trial-data-sharing.pdf?ver=2020-05-21-132838-960

## Abbreviations

aGVHD: Acute graft-versus-host disease
ALL: Acute lymphocytic leukemia
AML: Acute myeloid leukemia
ATG: Antithymocyte globulin
CMV: Cytomegalovirus
DLI: Donor lymphocyte infusion
DRI: Disease risk index
EBMT: European Society for Blood and Marrow Transplantation
EBMT–NIH-CIBMTR: European Society for Blood and Marrow Transplantation and National Institutes of Health Center for International Blood and Marrow Transplant Research
eCRFs: Electronic case report forms
GI: Gastrointestinal
GVHD: Graft-versus-host disease
H&OV: Hospital stays and outpatient visits
HLA: Human leukocyte antigen
HSCT: Hematopoietic stem cell transplantation
IBMTR: International Blood and Marrow Transplant Research
ICU: Intensive care unit
MAC: Myeloablative conditioning
MAGIC: Mount Sinai Acute GVHD International Consortium
MDS: Myelodysplastic syndrome
MPN: Myeloproliferative neoplasm
NHL: Non-Hodgkin lymphoma
PBSC: Peripheral blood stem cell
PD: Progressive disease
RIC: Reduced intensity conditioning
RWD: Real-world data
TBI: Total body irradiation
USD: US dollars
