# Supplementary material for: Burden of hospitalizations and outpatient visits associated with moderate and severe acute graft-versus-host disease in Finland and Sweden: a real-world data study
Source: Support Care Cancer. 2022 Mar 2;30(6):5125–35. doi: 10.1007/s00520-022-06915-9 (PMC9046314; doi:10.1007/s00520-022-06915-9)
Supplement: Supplementary file 1 — Supplementary file1 (DOCX 919 KB) [file 520_2022_6915_MOESM1_ESM.docx]

**Burden of Hospitalizations and Outpatient Visits Associated With Moderate and Severe Acute Graft-Versus-Host Disease in Finland and Sweden: A Real-World Data Study**

Lorenzo Sabatelli, PhD,^1^ Mikko Keränen, MD, PhD,^2^ Elisabet Viayna, PhD,^3^ Monserrat Roset, BSc Stats,^3^ Nuria Lara, MD, MSc,^3^ Daniel Thunström, MSc Pharm,^1^ Minja Pfeiffer, PhD,^1^ Malin Nicklasson, MSc Pharm,^4^ Maija Itälä-Remes, MD, PhD^5^

^1^Incyte Biosciences International Sàrl, Morges, Switzerland; ^2^Helsinki University Hospital, Helsinki, Finland; ^3^IQVIA Real World Solutions, Barcelona, Spain; ^4^Department of Hematology and Coagulation, Sahlgrenska Academy, University of Gothenburg, Gothenburg, Sweden; ^5^Turku University Hospital, Turku, Finland

**Online Resources**

**Contents**

[Online Resource 1 Study design. 2](#_Toc89176126)

[Online Resource 2 Comparison of (A) aGVHD severity grading and (B) organ staging across scales. 3](#_Toc89176127)

[Online Resource 3 Mapping rules for comparing MAGIC grading to other scales. 5](#_Toc89176128)

[Online Resource 4 Overview of aGVHD grading systems. 6](#_Toc89176129)

[Online Resource 5 Acute GVHD severity at diagnosis, based on MAGIC and mGlucksberg criteria. 7](#_Toc89176130)

[Online Resource 6 Length of hospital stay. 8](#_Toc89176131)

[Online Resource 7 Description of grading mapping used within the study 9](#_Toc89176132)

[Online Resource 8 Estimated costs for hospitalizations and outpatient visits for patients with aGVHD, based on average costs in Finland and Sweden. 11](#_Toc89176133)

# **
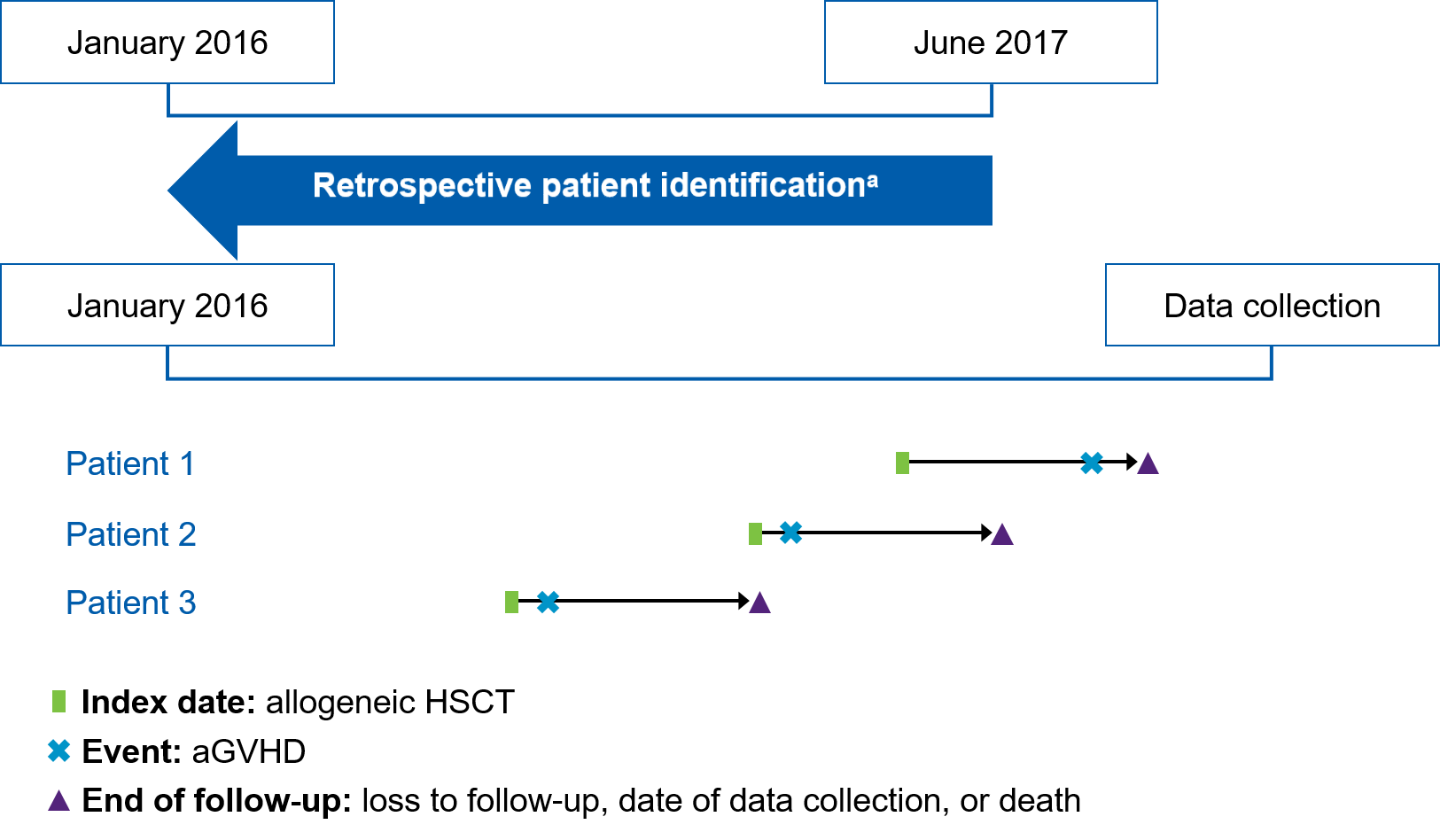
Online Resource 1** Study design.

aGVHD, acute graft-versus-host disease; HSCT, hematopoietic stem cell transplantation.
^a^ Patients were included retrospectively and consecutively, starting with those who received HSCT on June 30, 2017 and subsequently developed aGVHD, and working backward recruiting those who had received HSCT until January 1, 2016, or until the target sample size of approximately 4 to 25 patients per center had been reached, whichever occurred first.

#
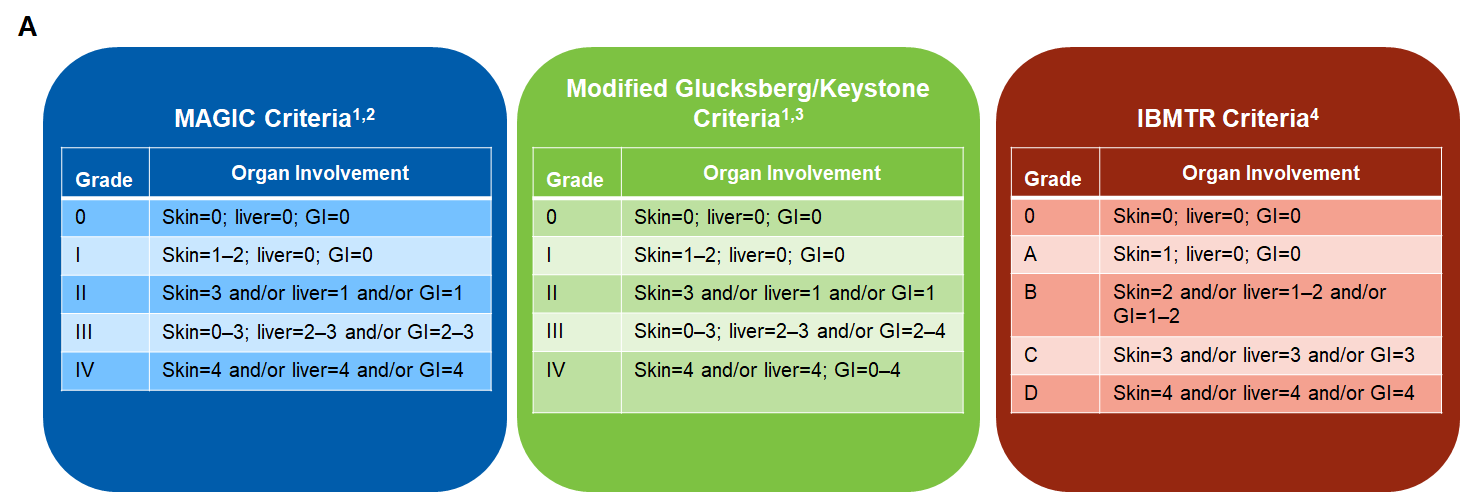
**Online Resource 2** Comparison of (A) aGVHD severity grading and (B) organ staging across scales.


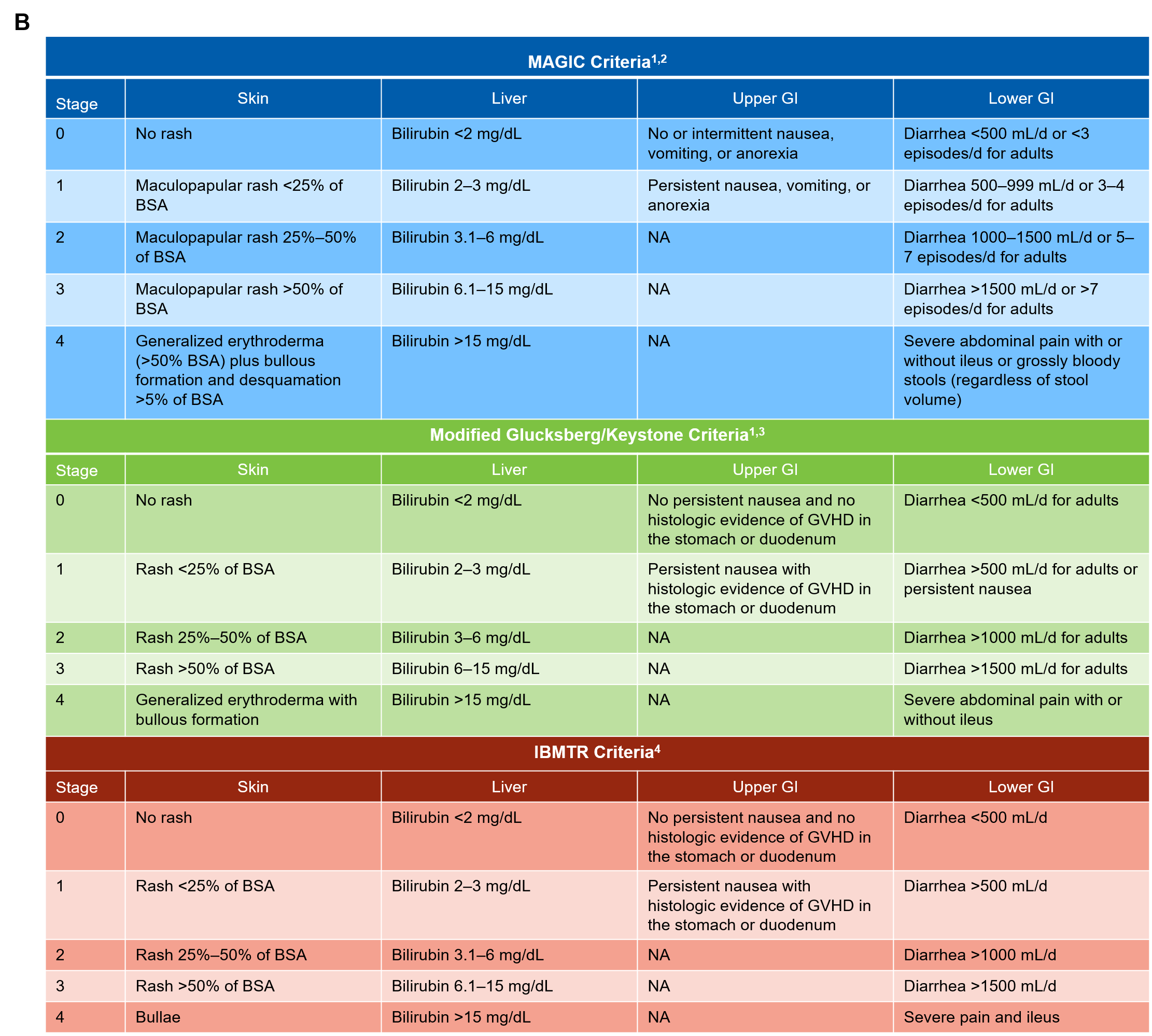
aGVHD, acute GVHD; BSA, body surface area; GI, gastrointestinal; GVHD, graft-versus-host disease; IBMTR, International Blood and Marrow Transplant Research; MAGIC, Mount Sinai Acute GVHD International Consortium; NA, not applicable.
1. Schoemans HM, et al. EBMT-NIH-CIBMTR Task Force position statement on standardized terminology & guidance for graft-versus-host disease assessment. *Bone Marrow Transplant*. 2018;53(11):1401-1415.
2. Harris AC, et al. International, multicenter standardization of acute graft-versus-host disease clinical data collection: a report from the Mount Sinai Acute GVHD International Consortium. *Biol Blood Marrow Transplant*. 2016;22:4-10.
3. Przepiorka D, et al. 1994 Consensus conference on acute GVHD grading. *Bone Marrow Transplant*. 1995;15:825-828.
4. Rowlings PA, et al. IBMTR Severity Index for grading acute graft-versus-host disease: retrospective comparison with Glucksberg grade. *Br J Haematol*. 1997;97:855-864.

# **
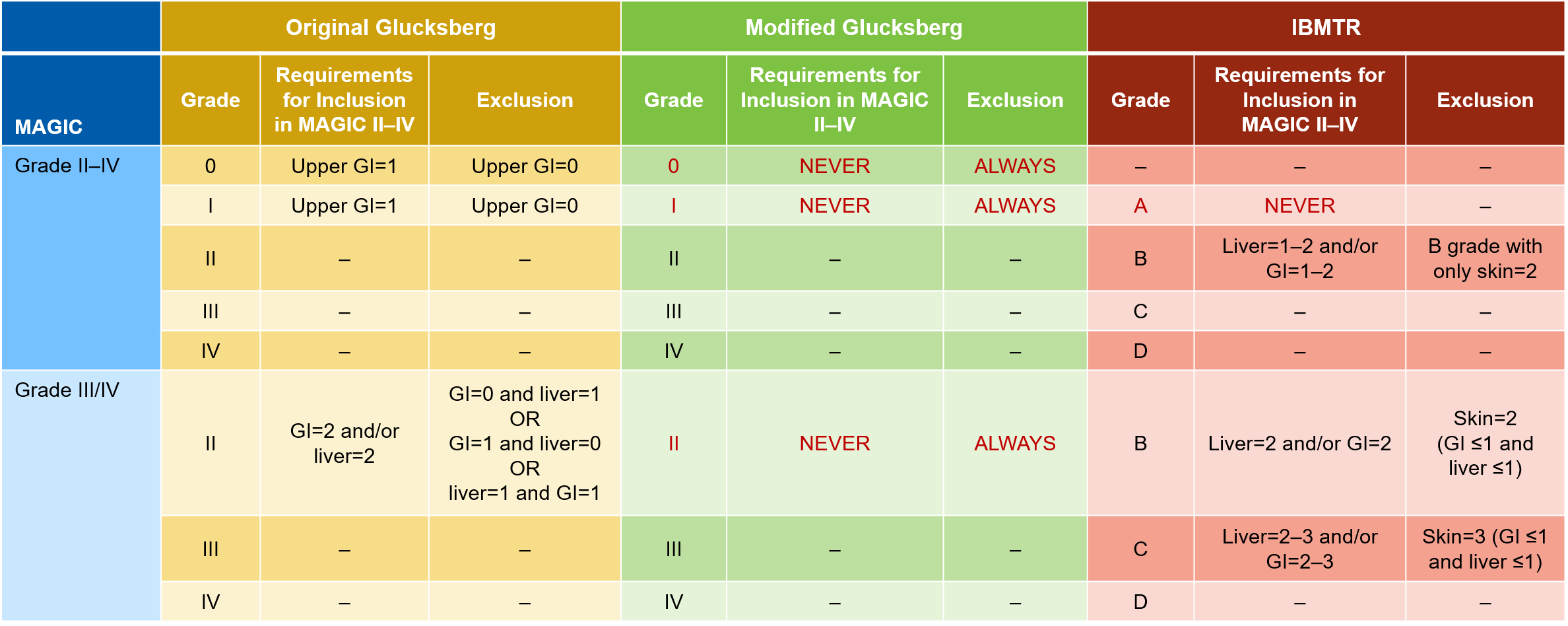
Online Resource 3** Mapping rules for comparing MAGIC grading to other scales.

GI, gastrointestinal; GVHD, graft-versus-host disease; IBMTR, International Blood and Marrow Transplant Research; MAGIC, Mount Sinai Acute GVHD International Consortium.

# **
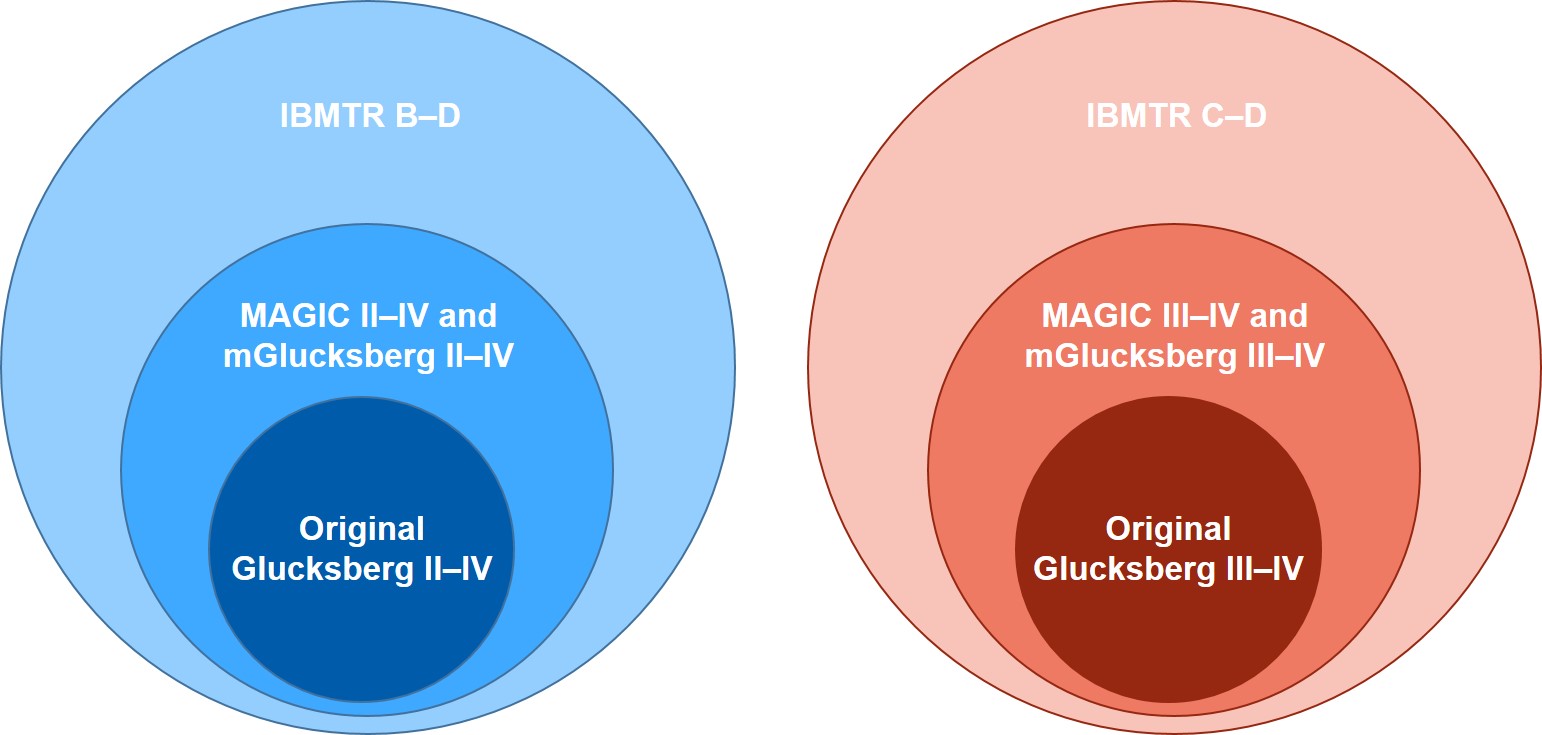
Online Resource 4** Overview of aGVHD grading systems.

aGVHD, acute graft-versus-host disease; IBMTR, International Blood and Marrow Transplant Research; MAGIC, Mount Sinai Acute GVHD International Consortium; mGlucksberg, modified Glucksberg.

# **
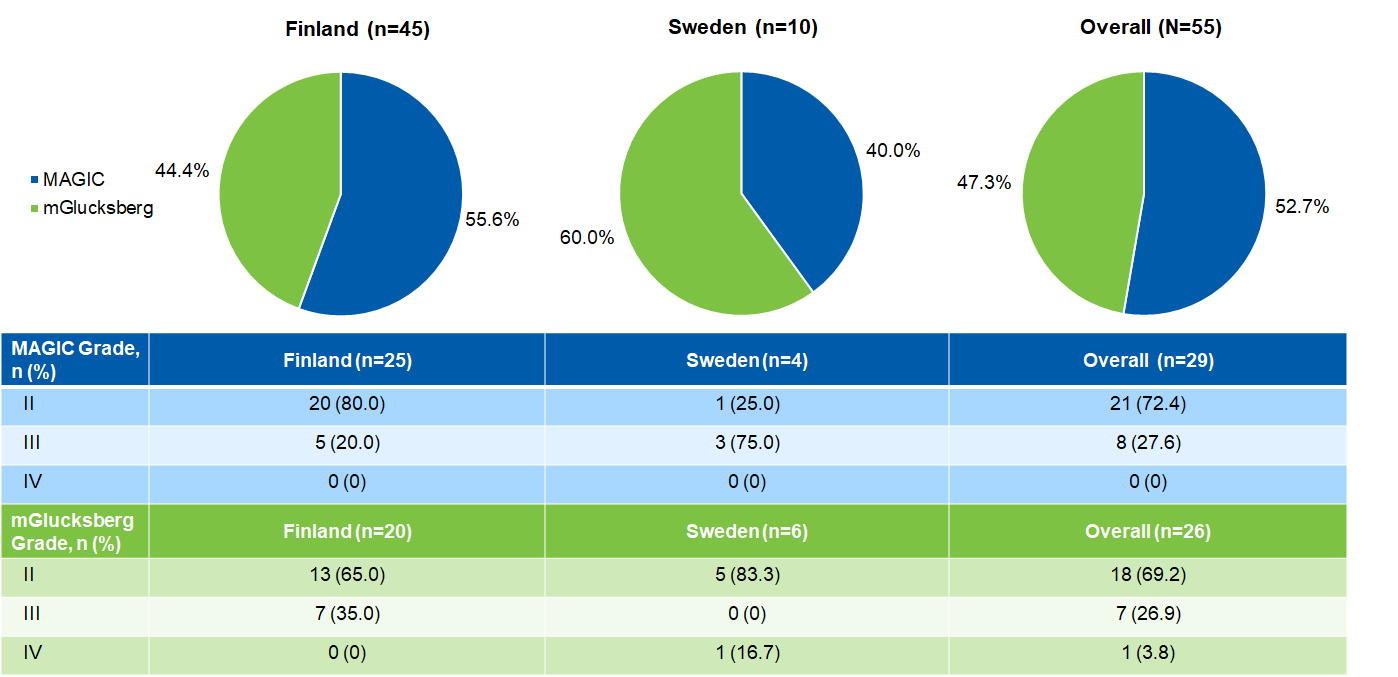
Online Resource 5** Acute GVHD severity at diagnosis, based on MAGIC and mGlucksberg criteria.

GVHD, graft-versus-host disease; MAGIC, Mount Sinai Acute GVHD International Consortium; mGlucksberg, modified Glucksberg.

# **
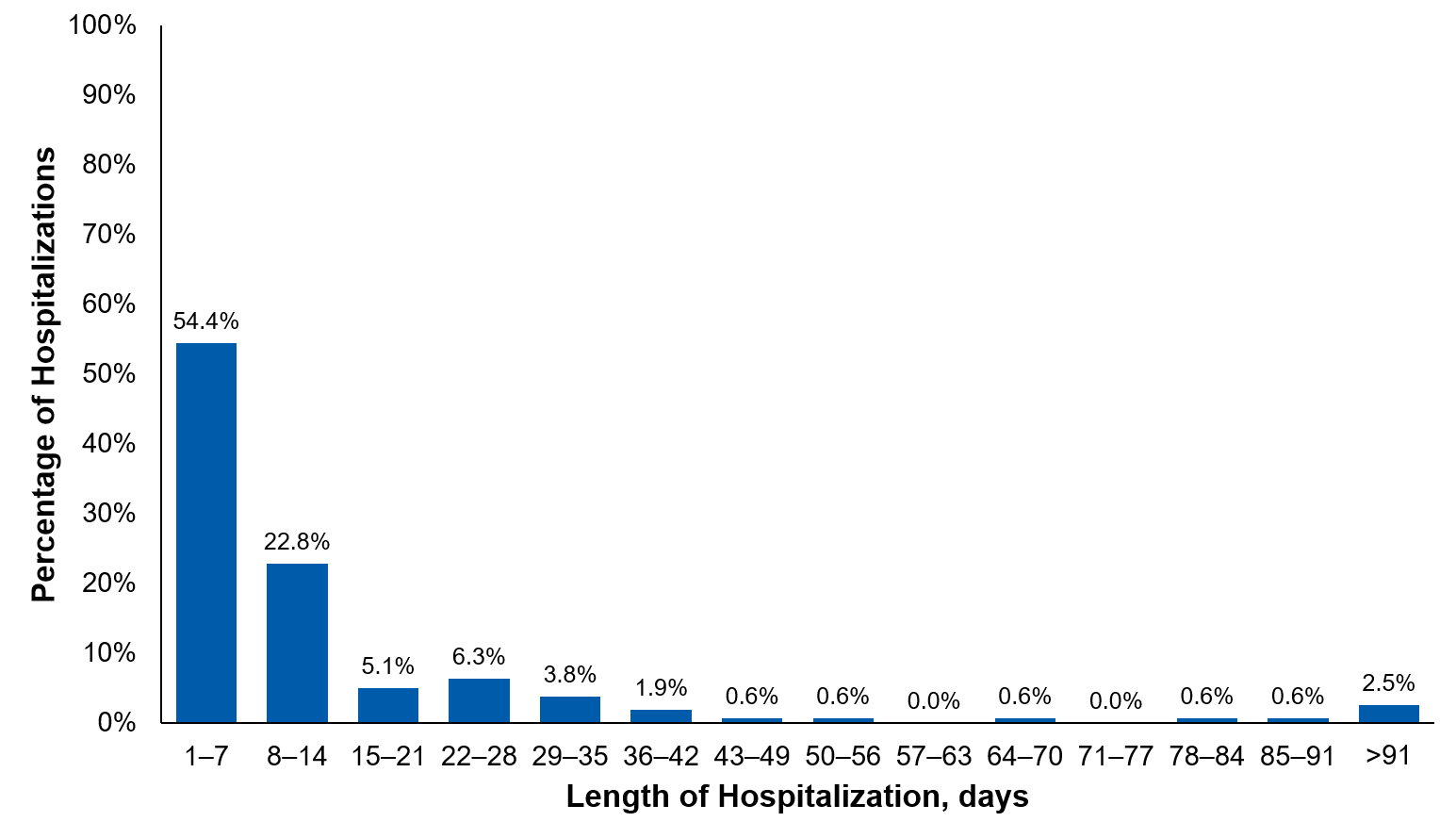
Online Resource 6** Length of hospital stay.

# **Online Resource 7** Description of grading mapping used within the study

The definitions of aGVHD grades and organ stages are reported in **Online Resource 2**. From those definitions, we can infer that:

- The main differences between the staging systems used for the MAGIC vs modified Glucksberg criteria are in the criteria used for gastrointestinal (GI) tract involvement
- For upper GI involvement, unlike MAGIC, modified Glucksberg also requires histological evidence of aGVHD in the stomach and duodenum. This means that while stage 1 upper GI involvement in modified Glucksberg is definitely stage 1 per MAGIC, some cases of stage 1 upper GI involvement in MAGIC may potentially be stage 0 in modified Glucksberg
- For lower GI, the main difference is that MAGIC staging can be based either on volume or on frequency of diarrhea. If volume is used, the 2 staging systems are almost identical (with the caveat that lower GI involvement may be considered stage 4 by MAGIC criteria in the presence of grossly bloody stools). However, if frequency of diarrhea is used for staging by MAGIC criteria, there is the risk that the same patients would be classified as having a different lower GI involvement stage (typically 1 stage up or down) in the corresponding modified Glucksberg
- Another potential source of difference arises from the definition of stage 4 skin involvement. In MAGIC, a skin stage 4 involvement requires desquamation in >5% of body surface area in addition to generalized erythrodema with bulleous formation, which defines skin stage 4 in the modified Glucksberg scale. Therefore, some skin stage 4 involvement in modified Glucksberg may be classified as a skin stage 3 involvement in MAGIC in the absence of significant desquamation, implying that some grade IV patients in modified Glucksberg would be classified as grade III in MAGIC
- If organ involvement staging was perfectly consistent across scales, grades 0, I, and II would be the same for MAGIC and modified Glucksberg scales. However, grades III and IV would still be differently defined in the two scales. In MAGIC, stage 4 GI involvement always leads to the assignment of grade IV aGVHD, whereas in modified Glucksberg it leads to the assignment of grade IV only if accompanied by stage 4 for skin and/or liver involvement
- If grade III and IV are grouped together, there are no differences between grades III/IV in MAGIC or modified Glucksberg, assuming staging is the same. However, the difference in the definition of GI stages may still generate uncertainty on whether a MAGIC grade II is a modified Glucksberg grade II or III and whether a MAGIC grade III is a modified Glucksberg grade II, III, or IV. Conversely, grade II per modified Glucksberg scale would be still classified as a grade II per MAGIC, and grade III per modified Glucksberg scale would be classified as a grade III or IV per MAGIC

The differences in GI and skin involvement staging, and in the grade definitions across the two scales, suggest that modified Glucksberg grade II can be converted into MAGIC grade II and that modified Glucksberg grade III/IV can be converted into MAGIC III/IV, with low risk of bias (see **Online Resources 3** and **4**).

Therefore, when converting modified Glucksberg grades into MAGIC grades, a group of patients with grade II aGVHD per modified Glucksberg can still be considered grade II per MAGIC, and a group of patients with grades III/IV per modified Glucksberg can be considered grade III/IV per MAGIC. This equivalence proves particularly useful when comparing population outcomes, rather than individual patient outcomes.

# **Online Resource 8** Estimated costs for hospitalizations and outpatient visits for patients with aGVHD, based on average costs in Finland and Sweden.

|  | Finland (n=45) | | | Sweden (n=10) | | |
| --- | --- | --- | --- | --- | --- | --- |
| Type of Cost | Mean Number of Days or Visits per Patient | Average Cost of Visit | Average Cost per Patient | Mean Number of Days or Visits per Patient | Average Cost of Visit | Average Cost per Patient |
| Hospitalization | 31.8 | $830–$1200^a^ | $26,394–$38,160 | 83.0 | $1100^c^ | $91,300 |
| Outpatient visit | 9.3 | $60–$180^b^ | $558–$1674 | 23.7 | $200^d^ | $4740 |

aGVHD, acute graft-versus-host disease; SEK, Swedish krona; USD, United States dollar.

^a^ Calculated based on average costs of 1 day at the transplantation unit in Turku (€700 or ~$830 USD) and Helsinki (€995 or ~$1200 USD), multiplied by the mean number of hospitalization days per patient.

^b^ Calculated based on average costs of 1 outpatient visit in Turku and Helsinki (€50–€150 or ~$60–$180 USD), multiplied by the mean number of outpatient visits per year per patient.

^c^ Calculated based on average costs of 1 day at the transplantation unit in Gothenburg (9514 SEK or ~$1100 USD), multiplied by the mean number of hospitalization days per patient.

^d^ Calculated based on average costs of 1 outpatient visit in Gothenburg (1735 SEK or ~$200 USD), multiplied by the mean number of hospitalization days per patient.
